# Association between alexithymia, emotional intelligence, smoking addiction, and alcohol use disorder among a sample of Lebanese adults

**DOI:** 10.1371/journal.pone.0295114

**Published:** 2023-11-30

**Authors:** Lara Youssef, Pascale Salameh, Hala Sacre, Marwan Akel, Souheil Hallit, Sahar Obeid

**Affiliations:** 1 Modern Dagher Medical Laboratories, Batroun, Lebanon; 2 School of Medicine, Lebanese American University, Byblos, Lebanon; 3 Institut National de Santé Publique, Epidémiologie Clinique et Toxicologie (INSPECT-LB), Beirut, Lebanon; 4 Department of Primary Care and Population Health, University of Nicosia Medical School, Nicosia, Cyprus; 5 Faculty of Pharmacy, Lebanese University, Hadat, Lebanon; 6 Drug Information Center, Order of Pharmacists of Lebanon, Beirut, Lebanon; 7 School of Pharmacy, Lebanese International University, Beirut, Lebanon; 8 School of Medicine and Medical Sciences, Holy Spirit University of Kaslik, Jounieh, Lebanon; 9 Research Department, Psychiatric Hospital of the Cross, Jall-Eddib, Lebanon; 10 Applied Science Research Center, Applied Science Private University, Amman, Jordan; 11 Social and Education Sciences Department, School of Arts and Sciences, Lebanese American University, Byblos, Lebanon; University of Botswana, BOTSWANA

## Abstract

**Background:**

Alexithymia can be associated with worse addictive traits, while emotional intelligence is associated with better addictive outcomes. In Lebanon, the prevalence of cigarette and waterpipe smoking is on the rise, although people are aware of the associated harms. Also, around 11% of Lebanese adults have experienced alcohol use disorder (AUD). This study aimed to assess the association between alexithymia, emotional intelligence, smoking (cigarette and waterpipe), and AUD among a sample of Lebanese adults.

**Methods:**

A web-based cross-sectional study carried out between February and April 2020, during the lockdown period, enrolled 408 community-dwelling adults. The survey link was shared on social media to reach participants from all Lebanese districts/governorates.

**Results:**

Taking antidepressants (Beta = 4.37) was significantly associated with more cigarette dependence, while female gender (Beta = -1.52) and having a high vs. low monthly income (Beta = 1.02) were significantly associated with less cigarette dependence. None of the variables, including alexithymia, were significantly associated with waterpipe dependence. Female gender (Beta = -0.15) and higher emotional intelligence (Beta = -0.003) were significantly associated with less AUD, whereas higher alexithymia (Beta = 0.003) was significantly associated with more AUD.

**Conclusion:**

This study could demonstrate a significant association between alexithymia and cigarette smoking and alexithymia and alcohol consumption. Future research is warranted to investigate the mediating effect of emotional intelligence and how these results may be used to meet the needs of alexithymic individuals with addictions.

## Background

The concept of addiction has been widely debated to portray a wide range of behaviors [1–4], including cravings, loss of control, or persistence despite the adverse physical, mental, and social consequences [5, 6]. Recently, addiction was defined as a chronic condition that affects the motivational system, where an irregular and possibly damaging high priority is given to a particular subject or activity [7].

Cigarette and waterpipe smoking and alcohol use disorder are three types of addictions, among several others, with a high prevalence in society [8–10]. The degree of addiction depends on several factors. For example, cigarette smoking is related to gender, age, depression, and anxiety [8], while waterpipe smoking is associated with female gender and older age [11, 12]. Regarding alcohol use disorder, it is correlated with anxiety, antisocial personality disorder, and depression [11, 13].

These addictions, particularly cigarette smoking, are associated with the use of anxiolytics, antipsychotics, and antidepressants [14, 15]. This association is primarily attributed to the fact that individuals trying to quit smoking or even drinking rely on anxiolytics and antidepressants to help them with withdrawal symptoms [16, 17]. A study among US college students revealed that anxiolytic users were four times more likely to be cigarette smokers [18]. Also, since the association between alcohol addiction and depression is already established, it is hypothesized that an association exists between antidepressant intake and alcohol consumption [16, 19].

These addictions are influenced by socioeconomic factors and mental disorders [8, 11, 13], including alexithymia, which has been investigated to check for association with increased addiction [20]. First defined by Sifneos in 1973 as the difficulty in identifying and communicating emotions [21], alexithymia is a state of an emotional deficit where individuals find it hard to process and regulate their feelings [22]. More precisely, it is the inability of cognitive processing of emotions, accompanied by the reduced capacity to recognize and express emotions [23]. Based on this definition, the literature has linked emotional dysregulation in alexithymia to the lack of emotional control in addiction [24]. Conflicting theories suggest that alexithymia could be either a vulnerability factor in mental disorders or a protective mechanism against psychological distress [25]. In both cases, this emotional deficit can play a crucial role in initiating and developing addictions among adolescents and adults. Thus, alexithymia facilitates the development of substance dependence [26]. Indeed, previous findings showed high levels of alexithymia in persons addicted to alcohol and smoking [20].

The association between alexithymia, smoking, and alcohol abuse is influenced by some demographic factors like age and gender. For example, alexithymic men are usually more prone to alcohol addiction than women [20, 27], while no difference is found between genders regarding nicotine addiction [28]. Also, older age is associated with higher alcohol or nicotine dependence [20].

In addition to the association of alexithymia with addictive practices, the literature mentions emotional intelligence (EI) as a dimension that can control or affect addiction [29, 30]. EI is defined as the ability to monitor own and others’ feelings and emotions, discriminate between this information, and use it to guide thinking and decisions [31]. It covers four dimensions, i.e., perception, understanding, management, and use of emotions [32, 33]. Therefore, it is hypothesized that people with higher EI have better control of their lives and better health outcomes. Moreover, higher EI is associated with better control of impulses and better management of addictions [30], while lower levels of EI can be associated with higher alcohol use disorder and smoking.

Decoding and differentiation of emotions and regulation of emotions are the two components of EI. These two components play a vital role in regulating and controlling addictions [29]. It is essential to distinguish between the different elements of emotions when studying the regulation of addiction. While higher alexithymia can worsen addictions, higher EI can decrease its intensity [29].

The negative reinforcement model elucidates how substance use leads to reduced stress levels of users, thereby increasing the likelihood of future substance reuse and the development of dependence [34, 35]. Similarly, another theory posits that addiction is fueled by the emotional drive to alleviate negative affect, thus creating a vicious cycle and interconnection between emotions and addictions. Several models have explained this relationship in the literature, but the main robust link between the two is the negative reinforcement effect [34].

### Theoretical framework, rationale, and hypothesis

According to the transdiagnostic theoretical framework introduced by Leventhal and Zvolensky, transdiagnostic emotional vulnerabilities link various anxiety and depressive psychopathologies to smoking [36]. Here, the transdiagnostic emotional vulnerabilities are presented by anxiety sensitivity (fear of anxiety), anhedonia (interest in response to reward), and distress tolerance (ability to withstand distressing states), which are the core behavioral traits responsible for maladaptive responses and emotional psychopathology. This framework posits that interest in reward amplifies smoking habits and thus the pleasure-enhancing effect, that anxiety sensitivity intensifies the anxiolytic effect of smoking, and that distress tolerance increases the distress-terminating effect of smoking. These three processes reinforce smoking among people with emotional psychopathology, increasing the risk of initiating smoking, sustaining the behavior over time, hindering cessation efforts, and contributing to relapse [36].

Moreover, it is well-established that alexithymia is associated with worse addictive traits and that EI relates to better addictive outcomes, hence the importance of highlighting and focusing on alexithymia and EI when treating addictions. In Lebanon, the population has endured several stressful situations due to numerous political instabilities [37, 38], which made the Lebanese population more susceptible to mental health disorders [39–43]; the latter may be underreported, mainly due to the prevalent cultural norms and taboo of seeking professional support. This social stigma towards seeking mental health support made the Lebanese population more prone to substance use and misuse as a coping mechanism [44–46]. Previous literature has shown a high prevalence of substance abuse among Lebanese adults [47]. Indeed, in Lebanon, the prevalence of cigarette and waterpipe smoking is on the rise due to the misperception of harm and inadequate knowledge [48–51]. Between 1999 and 2007 alone, waterpipe smoking increased by 200% and 60% among women and men, respectively [52]. Approximately 11% of adults in Lebanon have encountered alcohol use disorder, while a staggering 87% of the Lebanese population initiated alcohol consumption before reaching the age of 14 [47]. However, there is little information in the literature on alcohol use disorders, partly due to cultural and religious restrictions in Lebanon and the Arab world in general [44].

Based on the above theoretical framework and current evidence in Lebanon, it is hypothesized that Lebanese adults with alexithymia and lower EI would have higher cigarette dependence, waterpipe dependence, and alcohol use disorder. It is also assumed that gender and age might play a significant role in influencing these associations, with being a male and of older age being linked to higher levels of dependency. However, the main challenge in understanding this matter stems from the insufficient evidence regarding the relationship between these behaviors and emotional disorders. Here, our study comes into play, aiming to investigate the correlation between these and provide an analysis of the underlying association. Given the detailed evidence highlighting the correlation between alexithymia and the neural functions related to loss, pleasure, and reward, and considering the complex consequences of war on mental health in Lebanon and the societal taboo surrounding this issue, this study will explore the abovementioned variables in the Lebanese population. Therefore, this study aimed to assess the association between alexithymia, emotional intelligence, cigarette and waterpipe dependence, and AUD among a sample of Lebanese adults.

## Methods

### Ethics approval and consent to participate

The Psychiatric Hospital of the Cross Ethics and Research Committee approved the study protocol (HPC-020-2020). The purpose and requirements of the study were explained to each participant; filling the form and submitting it online was considered as giving a consent to participate in the study.

### General study design

A web-based cross-sectional study carried out between February and April 2020 during the COVID-19 lockdown imposed by the government enrolled a convenient sample of 408 community-dwelling smoking adults using the snowball technique. Due to commuting restrictions, a survey was created on Google Forms, and the link was shared on social media to reach participants from all Lebanese districts/governorates (Beirut, Mount Lebanon, North Lebanon, South Lebanon, and Beqaa). All individuals above 18 years and living in Lebanon were eligible to participate.

The self-administered questionnaire with closed-ended questions was anonymous and available in Arabic. The estimated time for completion was 25–30 minutes. The scales were forward and back-translated. Forward translation (English to Arabic) and back-translation were performed by two different translators. Minor discrepancies were solved by consensus.

### Sample size calculation

The G-power software calculated a minimum sample of 395 participants, based on an effect size f2 = 2%, an alpha error of 5%, a power of 80%, and considering 12 factors to be entered in the multivariable analysis.

### Questionnaire and measures

The questionnaire consisted of different sections. The first part clarified sociodemographic characteristics (age, gender, geographic region, marital status, number of children, work status, and education level). It also included information about cigarette smoking and monthly household income, divided into four categories: no income, low income (< 1000 USD), intermediate income (1000–2000 USD), and high income (> 2000 USD). Questions regarding the intake of anxiolytics/sedatives, antidepressants, and antipsychotics were also included in the survey.

The second part of the questionnaire comprised the following scales:

#### Toronto Alexithymia Scale (TAS 20)

Validated in Lebanon [53], the TAS used to assess alexithymia [54, 55] is rated on a 5-point Likert scale, with higher scores reflecting more alexithymia (α_Cronbach_ = 0.929). It includes three sub-scales: difficulty identifying feelings (DIF: 7 items) (e.g., *I am often confused about what emotion I am feeling*—item 1), difficulty describing feelings (DDF: 5 items) (e.g., *It is difficult for me to find the right words for my feelings*—item 2), and externally oriented thinking (EOT: 8 items) (e.g., *I prefer to analyze problems rather than just describe them*—item 5).

#### Lebanese Waterpipe Dependence Scale-11 (LWDS11)

This tool includes 11 items measured on a 4-point Likert scale from 0 to 3 to assess waterpipe dependence (e.g., LWDS 1. *Number of times you could stop waterpipe for 7 days*?). The total score is calculated by summing the 11 items [56, 57]. Higher scores indicate higher waterpipe dependence (α_Cronbach_ = 0.607).

#### Fagerström Test for Nicotine Dependence (FTND)

This scale consists of six items, three dichotomous (yes/no) rated 0 and 1, and three multiple-choice questions measured from 0 to 3 (e.g., FTND1—*How soon after waking do you smoke your first cigarette*?). The higher the total score, the more intense the physical nicotine dependence [58] (α_Cronbach_ = 0.843).

#### Alcohol Use Disorders Identification Test (AUDIT)

Validated in Lebanon [59], this tool consists of ten items to assess alcohol use, drinking patterns, and alcohol-related issues (e.g., AUDIT1—*How often do you have a drink containing alcohol*?). It can be administered by a clinician or self-administered [60]. Scores of 8 or above indicate AUD, while scores below 8 indicate low AUD risk (α_Cronbach_ = 0.917).

#### Assessing Emotions Scale

Validated in Lebanon [61], this 33-item tool is a self-scoring questionnaire used to measure emotional intelligence [62] (e.g., *I know when to speak about my personal problems to others*—item 1). Higher scores indicate higher emotional intelligence (α_Cronbach_ = 0.975).

### Statistical analyses

Data were analyzed on SPSS software version 23. There were no missing values since all questions were required. Cronbach’s alpha value was recorded for reliability analysis of each scale. The AUDIT score was not normally distributed (skewness and kurtosis outside the -2 and +2 interval); the log transformation of the score was applied, which had a normal distribution since the skewness and kurtosis values varied between -2 and +2 [63]. The FTND and LWDS had a normal distribution following the same rule. These conditions consolidate the assumptions of normality in samples bigger than 300 [64]. Accordingly, the Student t-test was used to check for an association between both scores and dichotomous variables (i.e., gender and marital status), while the ANOVA test was used to compare between three or more means (i.e., education level and monthly income). Pearson correlation test was used to correlate two continuous variables. Stepwise linear regressions were conducted, taking the log AUDIT, FTND, and LWDS scores as the dependent variables, respectively. Covariates included in the multivariable models were those that showed significant associations with the dependent variables in the bivariate analysis. Values of p<0.05 were considered significant.

## Results

### Descriptive statistics

Table 1 presents the sample demographics and descriptive characteristics. The mean age was 31.37 ± 13.23 years, and the majority were females (55.1%). The mean and standard deviation of the scales were as follows: FTND (5.54 ± 2.60), LWDS (14.95 ± 5.25), AUDIT (5.19 ± 5.97), TAS-20 (41.66 ± 13.52), and emotional intelligence (82.18 ± 28.49).

**Table 1:** Sociodemographic and other characteristics of the participants (N = 408).

Variable	N (%)
**Gender**	
Male	183 (44.9%)
Female	225 (55.1%)
**Monthly income**	
Low (<1000 USD)	221 (54.2%)
Intermediate (1000–2000 USD)	114 (27.9%)
High (>2000 USD)	73 (17.9%)
**Marital status**	
Single/widowed/divorced	260 (63.7%)
Married	148 (36.3%)
**Education level**	
Secondary or less	116 (28.4%)
University	292 (71.6%)
**Intake of anxiolytics and sedatives (yes)**	47 (11.5%)
**Intake of antidepressants (yes)**	17 (4.2%)
**Intake of antipsychotics (yes)**	7 (1.7%)
	Mean ± SD
**Age (in years)**	31.37 ± 13.23

### Bivariate analysis of factors associated with cigarette and waterpipe dependence

The mean cigarette dependence score was significantly higher in males, married participants, those with high income, and those taking sedatives and antidepressants. Additionally, older age and a higher number of children were significantly associated with higher cigarette dependence.

A higher mean waterpipe dependence score was significantly found among those with a primary/complementary education level, an intermediate monthly income, and those taking sedatives and antidepressants. Older age was significantly associated with higher waterpipe dependence (Tables 2 and 3).

**Table 2:** Bivariate analysis of categorical variables associated with alcohol use disorder and cigarette and waterpipe dependence.

Variable	Cigarette dependence	Waterpipe dependence	Alcohol use disorder
**Gender**			
Male	6.06 ± 2.01	15.32 ± 4.91	0.41 ± 0.48
Female	4.89 ± 3.07	14.73 ± 4.80	0.23 ± 0.35
*P*	**0.035**	0.055	**<0.001**
**Marital status**			
Single/divorced/widowed	5.23 ± 2.53	14.75 ± 5.02	0.28 ± 0.41
Married	6.04 ± 2.64	15.42 ± 4.54	0.37 ± 0.44
*P*	**0.024**	0.204	**0.034**
**Education level**			
Secondary or less	2.42 ± 3.42	15.87 ± 4.94	0.37 ± 0.49
University	1.69 ± 2.85	14.65 ± 4.79	0.29 ± 0.39
*P*	**0.042**	**0.022**	0.133
**Monthly income**			
Low (<1000 USD)	5.04 ± 2.32	14.31 ± 4.93	0.26 ± 0.39
Intermediate (1000–2000 USD)	6.00 ± 2.95	15.89 ± 4.76	0.32 ± 0.44
High (>2000 USD)	6.53 ± 2.43	15.67 ± 4.48	0.46 ± 0.48
*P*	**0.022**	**0.003**	**0.003**
**Intake of sedatives**			
No	5.30 ± 2.63	14.67 ± 4.83	0.30 ± 0.41
Yes	6.90 ± 1.95	17.49 ± 4.36	0.43 ± 0.48
*P*	**0.02**	**<0.001**	0.084
**Intake of antidepressants**			
No	5.33 ± 2.55	14.87 ± 4.85	0.30 ± 0.41
Yes	7.43 ± 2.24	18.00 ± 4.15	0.53 ± 0.58
*P*	**0.003**	**0.002**	0.136

**Table 3:** Bivariate analysis of continuous variables associated with cigarette and waterpipe dependence.

Variable	1	2	3	4	5	6
1. Alcohol use disorder	1					
2. Cigarette dependence	**0.22**	1				
3. Waterpipe dependence	**0.17**	0.04	1			
4. Age	**0.17**	0.08	**0.19**	1		
5. Alexithymia	**0.10**	0.07	0.07	**0.15**	1	
6. Emotional intelligence	**-0.21**	-0.04	-0.08	-0.08	0.04	1

^a^ p<0.001;

^b^ p<0.01;

^c^ p<0.05

### Multivariable analysis of factors associated with cigarette and waterpipe dependence

Correlations between the variables did not exceed 0.8, demonstrating sufficient discriminant validity for the measurement of the different factors examined in each model.

Antidepressant intake (Beta = 4.37) was significantly associated with more cigarette dependence, while female gender (Beta = -1.52) and having a high vs. low monthly income (Beta = 1.02) were significantly associated with less cigarette dependence (Table 4, Model 1).

**Table 4:** Multivariable analysis of factors associated with alcohol use disorder, cigarette and waterpipe dependence.

Variable	Unstandardized Beta	Standardized Beta	*p*	95% CI
**Model 1: Linear regression taking the cigarette dependence score as the dependent variable (R**^**2**^ **= 15%)**				
Gender (females vs males*)	-1.52	-0.25	**<0.001**	-2.10; -0.93
Sedatives intake (yes vs no*)	0.42	0.05	0.422	-0.61; 1.46
Antidepressants intake (yes vs no*)	4.37	0.29	**<0.001**	2.86; 5.88
Marital status (married vs single*)	0.63	0.10	0.076	-0.07; 1.33
Intermediate monthly income vs low*	-0.15	-0.02	0.673	-0.87; 0.56
High monthly income vs low*	-1.02	-0.13	**0.031**	-1.94; -0.10
Age	-0.01	-0.03	0.684	-0.04; 0.02
Alexithymia	0.01	0.02	0.615	-0.02; 0.03
**Model 2: Linear regression taking the waterpipe dependence score as the dependent variable (R**^**2**^ **= 6.1%)**				
Gender (females vs males*)	-0.35	-0.04	0.487	-1.33; 0.64
Sedatives intake (yes vs no*)	1.43	0.09	0.112	-0.33; 3.18
Antidepressants intake (yes vs no*)	1.57	0.07	0.226	-0.98; 4.11
Intermediate monthly income vs low*	0.74	0.07	0.231	-0.47; 1.94
High monthly income vs low*	0.25	0.02	0.755	-1.32; 1.82
Age	0.04	0.10	0.125	-0.01; 0.09
Alexithymia	0.01	0.03	0.598	-0.03; 0.05
Education level (university vs secondary or less*)	-0.11	-0.01	0.852	-1.30; 1.07
**Model 3: Linear regression taking the AUDIT score as the dependent variable (R**^**2**^ **= 10.9%)**				
Gender (females vs males*)	-0.15	-0.18	**<0.001**	-0.23; -0.07
Marital status (married vs single*)	0.06	0.07	0.243	-0.04; 0.16
Intermediate monthly income vs low*	0.002	0.002	0.972	-0.10; 0.10
High monthly income vs low*	0.06	0.05	0.391	-0.07; 0.19
Age	0.002	0.05	0.421	-0.002; 0.01
Alexithymia	0.003	0.10	**0.049**	0.001; 0.006
Emotional intelligence	-0.003	-0.19	**<0.001**	-0.004; -0.001

*Reference group; CI = Confidence Interval; numbers in bold refer to significant *p* values.

None of the variables, including alexithymia, was significantly associated with waterpipe dependence (Table 4, Model 2).

Female gender (Beta = -0.15) and higher EI (Beta = -0.003) were significantly associated with less alcohol use disorder, whereas higher alexithymia (Beta = 0.003) was significantly associated with more alcohol use disorder (Table 4, Model 3).

## Discussion

### Alexithymia and alcohol use disorder

Our results indicated that higher alcohol use disorder was associated with higher alexithymia. Evidence has shown the inability of alexithymic individuals to differentiate between different emotional states. Thus, the induced emotional state and mood-altering effect of alcohol consumption can rarely be achieved by alexithymic people, consequently increasing their alcohol consumption to reach this state [65]. Previous findings have described how heavy alcohol drinkers who also have alexithymia report obsessive urges and alcohol cravings [66]. Alcohol intoxication temporarily reduces the emotional dysfunction experienced by alexithymic persons, leading them to seek more alcohol to compensate for the emotional imbalance and enhance their ability to access their feelings [67].

Our study could not demonstrate an association between higher alexithymia and increased alcohol use, contradicting what is available in the literature. Indeed, people with alexithymia are usually emotionally unaware and develop substance dependence [20, 68], which generally translates into increased tobacco smoking or binge drinking [22, 68]. The literature has repeatedly reported that people with emotional illnesses, such as depression and alexithymia, are more vulnerable to responding to these emotions by engaging in addictive habits. Similar to the transdiagnostic theoretical framework suggested by Leventhal and Zvolensky, people with emotional psychopathologies intensify the risk of adopting smoking habits. These people are more likely to start smoking, sustain this habit, and recurrently try to avoid cessation [36].

Although evidence in the literature regarding the association between alexithymia and waterpipe smoking is scarce, our results revealed that alexithymia was not associated with waterpipe dependence, aligning with the findings of some studies that explored the relationship between alexithymia and smoking in general [69]. The addictive effects of waterpipe smoking are not appropriately perceived since people do not view the waterpipe to be as harmful as cigarettes [70, 71]. It is still unclear how mental health problems could be associated with waterpipe tobacco smoking, with many contradictory conclusions [69, 72].

### Sociodemographic characteristics and cigarette dependence

Our results showed that being a female was associated with lower cigarette dependence compared to being a male. In developing countries, the reported prevalence of cigarette smoking among women is often lower than among men, which matches our results and study location. Specifically, in Middle Eastern countries, females are less likely to use conventional methods of tobacco use, such as cigarette smoking [73]. The latter is perceived as a masculinity sign, which explains why being a woman might not be associated with cigarette dependence, while men are more likely to be smokers [74]. Also, the sexual context of a cigarette and the thought of it shaping men as more attractive leads to its higher association with the male gender [75]. Our data also suggest that having a lower monthly income was associated with higher cigarette smoking. Similarly, higher antidepressant intake was associated with higher cigarette smoking. Both factors are related to the environment in which participants live and their accessibility to tobacco retailers [76]. These results are consistent with those of a study in San Francisco, where people with mental illnesses lived in rural neighborhoods with twice as many tobacco stores [77].

### Emotional Intelligence and alcohol use disorder

Our study revealed that people with lower AUD had higher levels of EI. In other words, lower EI [78–80] is strongly associated with higher AUD. A possible reason is that people who excessively consume alcohol have poor emotional decoding skills and cannot identify emotions correctly [29]. Moreover, there is an additional negative association between alcohol use and the understanding and uttering of emotions [81]. Those who abuse alcohol struggle to comprehend and express their feelings. High alcohol consumption contributes to the ambiguity of people understanding their emotions, making them less aware of themselves and their emotions [82].

### Sociodemographic characteristics and alcohol use disorder

In this study, being a female was associated with lower AUD, consistent with findings from the literature and mainly explained by the social and cultural norms that dictate and influence gender differences in alcohol drinking. For example, men are free to drink in public, which rather reflects their superiority, without being judged as women are for the same behavior [78–80].

### Limitations

One of the principal limitations to consider in our study is social desirability bias, which means that some participants might have chosen to report positive behaviors, such as not smoking or drinking, affecting and skewing our results [83]. Moreover, our study is cross-sectional with relatively small sample size. Hence, our results are not generalizable, and there is a need for more rigorous longitudinal studies to deduce causation [84]. Additionally, the context of the pandemic also contributes to limiting our results generalizability. The mental health triggers that our sample experienced during the COVID-19 pandemic might have negatively affected their coping mechanism during lockdowns, thus increasing their alcohol and smoking consumption [85]. However, results from our study could serve for comparison with other research findings from different settings, contribute to the existing theoretical framework of knowledge of the topic, and develop and test new explanatory theories [86]. Participants might not have answered honestly and appropriately, which might lead to information bias. Selection bias is also possible due to the snowball sampling technique. The inclusion of a clinical population in our sample was not achievable but could be a prospect for future studies. Residual confounding bias is also possible since this study did not tackle all the factors that might be associated with smoking dependence and alcohol use disorder. Finally, the self-reported nature of the survey might have caused a selective recall bias, where participants tend to only report what they recognize as a risk factor or even under-report unhealthy behaviors [87].

## Conclusion

The results of our study will most likely prompt healthcare professionals to consider assessing psychological factors like alexithymia and emotional intelligence in individuals with substance use disorders [69]. Further studies are necessary to elucidate the relationship across these variables in Lebanon and suggest causality.
